# Induction chemotherapy with cetuximab, carboplatin and paclitaxel for the treatment of locally advanced squamous cell carcinoma of the head and neck

**DOI:** 10.3892/etm.2013.948

**Published:** 2013-02-05

**Authors:** JESSICA BAUMAN, COREY LANGER, HARRY QUON, KENNETH ALGAZY, ALEXANDER LIN, ARATI DESAI, FAITH MUTALE, JARED WEISS

**Affiliations:** 1 Division of Hematology and Oncology, Dana-Farber Cancer Institute, Boston, MA 02215;; 2 Division of Hematology and Oncology, University of Pennsylvania, Philadelphia, PA 19104;; 3 Department of Radiation Oncology, Johns Hopkins University, Baltimore, MD 21205;; 4 Department of Radiation Oncology, University of Pennsylvania, Philadelphia, PA 19104;; 5 Division of Hematology and Oncology, University of North Carolina, Chapel Hill, NC 27599, USA

**Keywords:** locally advanced squamous cell carcinoma of the head and neck, head and neck cancer, induction chemotherapy, chemoradiotherapy

## Abstract

Although controversy exists in the management of locally advanced squamous cell carcinoma of the head and neck (LA-SCCHN), clinicians often use induction chemotherapy for treatment of the most advanced cases. One promising regimen combines weekly cetuximab (400 mg/m^2^ loading dose followed by 250 mg/m^2^) with carboplatin (AUC of 2) and paclitaxel (90 mg/m^2^). We retrospectively evaluated patients treated with this regimen prior to definitive chemoradiation or surgery between May 2008 and December 2011. The primary endpoint used for this retrospective analysis was feasibility. Thirty consecutive, unselected patients were included. Median follow-up was 13.7 months (range, 5.0–38.7 months). All but one patient had stage IV SCCHN. Dose intensity was high for carboplatin (92%), paclitaxel (93%) and cetuximab (85%). Grade 3–4 toxicities occurred in <7% of the study population and were limited to rash, neutropenia and infusion reactions. Response rate (RR) to induction chemotherapy was 97% (30% complete response, 67% partial response). All patients completed subsequent chemoradiotherapy or surgery. Nineteen patients (63%) demonstrated a complete response and 11 patients (37%) demonstrated a partial response. Median overall survival and progression-free survival data are not yet mature. The RR to therapy in our off-protocol experience is at least comparable to that observed in the two phase II studies of this regimen and appears superior to that observed with docetaxel, cisplatin and fluorouracil (TPF).

## Introduction

The estimated incidence of squamous cell carcinoma of the head and neck (SCCHN) in the USA in 2010 was ∼49,260 cases with 11,480 mortalities (1). The majority of patients with newly diagnosed SCCHN present with locally advanced disease. Multiple treatment modalities have been utilized for locally advanced SCCHN (LA-SCCHN), including various combinations of chemotherapy, radiation therapy and surgery. However, the most effective combination has yet to be determined (2,3). When chemoradiotherapy is employed, the most commonly used standard regimen is daily radiation concurrent with three cycles of bolus cisplatin. Only bolus cisplatin and weekly cetuximab are supported by level I evidence (4).

Although chemoradiation has the potential to cure stage IV disease, a significant number of patients will relapse, particularly those with higher nodal status at presentation (5). Induction chemotherapy prior to definitive chemoradiation in patients with LA-SCCHN is one approach presently used to improve outcomes, although its use is controversial. Two phase III trials published in 2007, TAX 323 and TAX 324 (6,7), studied induction chemotherapy with docetaxel, cisplatin and fluorouracil (TPF) and provided indirect evidence of the efficacy of induction chemotherapy. The addition of docetaxel to cisplatin/fluorouracil in both studies increased response rates (RRs) as well as overall survival (OS) compared with that achieved with PF alone, however, neither study featured definitive standard of care chemoradiotherapy in the control arm. While these studies resulted in the FDA approval of docetaxel as part of induction chemotherapy in the USA, they were heavily criticized for comparing two experimental regimens, instead of comparing either to an accepted standard of care (8).

The high rates of toxicity reported in TAX 323 and TAX 324 led many oncologists to question the feasibility of TPF. In TAX 324, 21% of patients did not proceed to protocol-defined chemoradiotherapy and 7% of patients did not proceed to potentially curative therapy (7,9). Following the presentation of DeCIDE (10) and PARADIGM (11) trial data at ASCO 2012, enthusiasm for TPF diminished further. However, the ultimate utility of induction chemotherapy is yet to be resolved. Whether induction as a concept is flawed or whether it has been tested with the wrong regimen thus far remains unknown.

One of the most promising induction chemotherapy regimens evaluated to date consists of weekly cetuximab (C225), carboplatin and paclitaxel. Carboplatin and paclitaxel were used together in a phase II study consisting mainly of stage IV patients where a clinical complete RR of 100%, 3-year OS of 70% and a 3-year progression-free survival (PFS) of 90% were demonstrated following completion of definitive chemoradiotherapy (12). Although the regimen was less toxic compared with TPF, RR to induction chemotherapy was preserved; RR to TPF in TAX 323 and TAX 324 was 68 and 72%, respectively. In the study by Haraf *et al* (12), the RR to weekly carboplatin/paclitaxel was 82%. Contemporaneously, cetuximab was demonstrated to be efficacious in head and neck cancer yielding improvements in survival when used concurrently with radiation (13,14) and palliative chemotherapy (15). This led to interest in grafting cetuximab onto the carboplatin/paclitaxel induction backbone. This was first studied in a phase II clinical trial published in 2010 by Kies *et al* (16) as well as in a second phase II study by Wanebo *et al* (17). The Kies study employed six weekly doses of cetuximab, carboplatin and paclitaxel followed by radiation alone, chemoradiation or surgery using an adaptive risk-based strategy. All 47 patients proceeded to potentially curative therapy, and PFS and OS rates were 87 and 91%, respectively, following a median follow-up of 33 months. Response to induction was 96%. The Wanebo ‘organ preservation’ study included 74 operable patients with stage III or IV disease, each of whom were treated with weekly cetuximab, carboplatin and paclitaxel, followed by serial biopsies. In total, 65% of patients demonstrated a pathological complete response following induction and 100% demonstrated a pathological complete response following subsequent chemoradiation. Most recently, cetuximab, carboplatin and paclitaxel were evaluated with a different schedule, where carboplatin was administered on a monthly bolus (18). The RR to the induction portion of this study was 92%. The abstract did not report any toxicity from induction therapy alone, however, the total toxicity was high, which is likely based in part on the choice of subsequent chemoradiotherapy regimen.

Based upon these data, the treating clinicians at the University of Pennyslvania (PA, USA) viewed the combination of carboplatin, paclitaxel and cetuximab as a less toxic, potentially more efficacious alternative to TPF. The regimen was therefore adopted in 2008 as the exclusive induction strategy for advanced neck disease in SCCHN, typically N2b or greater.

## Patients and methods

### Patient identification

To be considered eligible for this retrospective review, patients had to have been diagnosed with LA-SCCHN between May 2008 and December 2011, and treated with the induction regimen of weekly cetuximab, carboplatin and paclitaxel prior to definitive chemoradiation or surgery. Appropriate patients were identified through review of all subjects treated during this period at the University of Pennsylvania with SCCHN. Patients who received this regimen in the setting of recurrent or metastatic SCCHN were excluded. None of these identified patients were excluded from the analysis.

### Database

Once patients were identified, we reviewed electronic medical charts to extract data for baseline characteristics including TNM status of the tumor, the primary tumor site, tumor HPV-16 status (if available), date of diagnosis, gender, performance status, Charlson Comorbidity Index, body weight throughout therapy and smoking status. Additionally, doses of induction chemotherapy as well as definitive chemotherapy and radiation regimens, date of treatment and toxicity of therapy were obtained. Toxicity was graded retrospectively by CTCAE version 3. These results were entered into a password-protected tumor database, which was maintained in Microsoft Access. This retrospective collection of data was approved by the University of Pennsylvania Institutional Review Board.

### Written informed consent

Written informed consent was not required as data were obtained retrospectively with the permission of the institutional review board. Patient data were de-identified upon chart extraction then maintained in a password-protected database. All patients receiving chemotherapy and cetuximab signed standard systemic therapy consent forms prior to receiving treatment.

### Data collection

Toxicity data were extracted retrospectively through chart review of EPIC, the outpatient electronic medical record used at the University of Pennsylvania. The data were gathered from review of oncology, nutrition and radiation oncology provider notes. All laboratory studies acquired subsequent to the initiation of chemotherapy and cetuximab were reviewed to assess for hematologic toxicity. All nonhematological toxicity data were obtained retrospectively only if they were recorded formally in a complete review of systems or through a standardized, itemized checklist used by the provider. If the data available were ambiguous in gradation of symptoms and severity, we defaulted to the higher grade of toxicity.

Treatment data were extracted from the outpatient EPIC system and through the inpatient order-entry system, Sunrise, when chemotherapy was administered on an inpatient basis. The nature and doses of chemotherapy were documented from scanned order sheets and electronic medical records. Doses of radiation were determined in the radiation oncology progress notes and in the summary completion notes. Reasons for changes in chemotherapy doses and regimens were documented in the oncology provider notes.

### Treatment

Induction chemotherapy with cetuximab, carboplatin and paclitaxel was most commonly administered over eight weeks, with an additional loading dose of 400 mg/m^2^ i.v. cetuximab administered alone the week prior to starting the three-drug regimen. During subsequent weeks, patients received cetuximab 250 mg/m^2^ in combination with weekly carboplatin AUC 2 and paclitaxel 90 mg/m^2^, with standard premedications of dexamethasone 12 mg i.v., diphenhydramine 50 mg i.v. and ondansetron 24 mg i.v. Treatment cycles were repeated weekly for up to 8 weeks and doses were withheld or modified at the provider’s discretion depending on the types of toxicities experienced.

Radiotherapy was initiated ∼3 weeks after induction chemotherapy was completed. Target volumes, duration of therapy and total radiation dose were determined by the treating radiation oncologist based on the standard of care. All radiation was planned for 7 weeks of the total treatment.

Concurrent therapy during radiotherapy was determined at the discretion of the provider. Depending on performance status and prior toxicities, the regimens used included either high-dose cisplatin (100 mg/m^2^) administered every 3 weeks for three doses, weekly cetuximab, weekly cisplatin (30 mg/m^2^) or weekly carboplatin (AUC 2) and paclitaxel (30 mg/m^2^). If toxicities were encountered during chemoradiotherapy, providers either reduced the dose of the chosen regimen or changed to a less toxic regimen including weekly cetuximab (250 mg/m^2^), weekly carboplatin (AUC 2) or weekly cisplatin (30 mg/m^2^). There were no prescribed criteria. No other agents were administered.

### Response

Partial or complete response to the combined induction chemotherapy and subsequent chemoradiotherapy or surgery was evaluated retrospectively through physical exam notation in provider notes, nasopharyngolaryngoscopy (NPL) where available and imaging (PET, CT or MRI). Partial and complete RRs were defined by the Response Evaluation Criteria in Solid Tumors (RECIST) criteria.

### Statistical analysis

Data from Microsoft Access were exported into Microsoft Excel where all analysis was completed. The primary endpoint of this retrospective analysis was feasibility, as measured by the number of induction cycles tolerated. Other pre-specified secondary endpoints included percentage of patients progressing to definitive chemoradiotherapy, toxicity, response and overall and median survival. Overall and median survival curves were calculated with the standard Kaplan-Meier method.

## Results

### Patients

Thirty patients were identified in this retrospective analysis between May 2008 and December 2011. These constituted all treatment-naïve patients receiving induction therapy for LA-SCCHN at the University of Pennsylvania during that period. Patient and tumor characteristics are summarized in Table I. The median age was 58.3 years (range, 37.4–83.6), median performance status was 0 and median Charlson Comorbidity index (combined condition and age-related score) was 4. Thirty percent were never smokers; of the smokers, 33% smoked ≤10 pack-years and 37% smoked >10 pack-years. Base of the tongue or tonsil were the primary tumor sites in 77% of the patients. Table II displays the tumor and nodal distribution matrix. No patient had metastatic disease at the start of induction chemotherapy. All but one patient had stage IVa or IVb SCCHN.

### HPV data

HPV data were available for 13 patients. p16 staining as a surrogate for HPV was reported as an addendum to pathology reports from patient specimens. Ten patients tested positive. The primary sites of these ten cases were all either base of tongue or tonsil. The primary sites of the three with negative p16 staining were base of tongue, hypopharynx and oral cavity.

### Treatment

**Figure 1:**
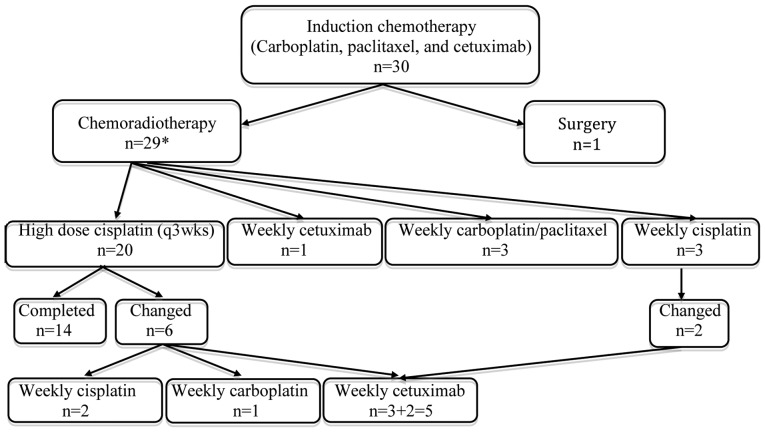
Patients’ treatment course. ^*^Of these 29 patients, 2 received chemoradiation at an outside institution and are not included in this figure.

All patients received induction chemotherapy with weekly paclitaxel, carboplatin and cetuximab, followed by combined chemoradiotherapy (n=29) or surgery (n=1). Treatment disposition is further delineated in Fig. 1. In addition, four patients underwent neck dissections due to possible residual disease on imaging.

### Induction chemotherapy and toxicity

The majority of patients received their intended treatment (Table III). Induction chemotherapy was administered over a median of 48.2 days (range, 13.2–53.7), with some variability in the number of pre-planned cycles. The most common planned regimen was eight cycles (not including the loading dose of 400 mg/m^2^ cetuximab, which all patients received the week prior to starting the three-drug regimen); however, the range was from 5 to 8 pre-planned cycles. The dose intensity for all treated patients was high and the number of doses withheld or reduced for all three agents was low (Table III).

Overall, induction chemotherapy was well tolerated (Table IV). There were no observed grade 5 events. The only grade 3–4 toxicities included rash (6.7%), neutropenia without fever (6.7%) and infusion reactions (3.3%). The most common grade 1–2 toxicities included rash (77%), nausea (37%), fatigue (37%), anemia (17%), alopecia (27%) and electrolyte abnormalities (13%). No renal toxicity, hearing loss or febrile neutropenia were observed. Three patients (10%) were hospitalized during induction chemotherapy; two for non-neutropenic infections and the third for neutropenia without fever.

The majority of patients’ body weights remained stable throughout induction chemotherapy; 3 weeks after induction, 23% of patients gained >5% of their initial body weight (10% gained >10%). Twenty-one patients experienced pain secondary to their cancer at the start of treatment and 15 of these patients (71%) had significant reduction or complete resolution of their cancer pain and/or dysphagia during their induction course.

### Response to induction chemotherapy

All patients were evaluated by imaging with CT, MRI and/or PET, physical examination and NPL prior to induction chemotherapy initiation. All were evaluated by physical examination and NPL post-induction, and 18 had imaging with MRI or PET. Using the medical record documentation of physical examination and NPL, and applying the RECIST criteria whenever possible, 9 patients (30%) had a complete clinical or radiographical response to induction chemotherapy, 20 patients (67%) had a partial response and 1 patient (3%) had stable disease. No patient experienced disease progression during induction chemotherapy.

### Chemoradiotherapy

Twenty-nine patients (97%) received chemoradiotherapy 3–4 weeks post-induction chemotherapy. All had completed their full treatment at the time of analysis. One patient went onto surgery due to prior head and neck radiation for lymphoma.

### Chemoradiotherapy and toxicity

Fig. 1 summarizes the treatment regimens of the 29 patients who completed chemoradiotherapy. Two patients received their chemoradiotherapy at an outside institution and thus their data were only partially analyzed. The median radiation dose administered was 7040 cGy (range, 2600–7040 cGy). Twenty-five of the 27 patients received 100% of their intended dose, one patient received 94% and one patient received only 37%. The median radiation course length was 6.7 weeks (range, 5.4–9.7 weeks). One patient (with a course length of 9.7 weeks) had a treatment interruption of 3 weeks due to non-compliance, although the patient ultimately received 100% of the intended radiation dose. Another patient (with a course length of 5.4 weeks) experienced several multiple day treatment interruptions and ultimately only received 2600 cGy (37%) of the intended dose due to non-compliance, despite multiple attempts to re-establish follow-up. The remaining 27 patients completed their radiation on schedule without interruption.

The initial planned concurrent chemotherapy regimen was changed in 9 patients (30%) due to toxicities (Fig. 1). The most common toxicities that required alteration of the regimen were hematological cytopenias and hearing loss. Table IV details the toxicity data for combined chemoradiotherapy. The most common grade 3 and 4 toxicities included mucositis, anorexia, odynophagia, neutropenia and thrombosis (67, 63, 41, 19 and 7%, respectively).

Weight loss was common during chemoradiotherapy compared with the induction phase of treatment; 59% of patients lost >5% of their baseline body weight and 22% lost >10% of their baseline body weight.

### Surgical outcomes

The one patient undergoing primary surgery instead of chemoradiotherapy for definitive therapy had tumor invading the submucosa on microscopic pathology (primary site; tonsil). Four other patients underwent neck dissections after they completed therapy due to concern for residual disease on imaging. Three of these patients had no evidence of disease on pathological examination. Pathological examination of one patient revealed microscopic foci of residual squamous cell carcinoma at a single lymph node level.

### Overall response

Of the 30 patients evaluated, all had completed their full treatment at the time of analysis. Of those 30, 29 underwent imaging post chemoradiotherapy, although the timing and type of imaging was variable. PET/CT was the most commonly used to follow treatment effect over time. In total, 27/30 patients underwent their first PET/CT between 2.5 months to 1 year after chemoradiation terminated. Applying the RECIST criteria to PET/CT, MRI or clinical exam, 19 (63%) had complete clinical or radiographical responses at 3–6 months post-treatment, and 18 of these patients continued to demonstrate no evidence of disease at their time of last follow-up (range, 5.0–38.7 months). One of these 19 patients sustained a local recurrence 7.5 months after completion of chemoradiation and has since succumbed to progression of disease. In addition, another one of the 19 patients succumbed to non-cancer-related issues. Of the 30 patients, 11 (37%) had partial responses at the end of full treatment with induction chemotherapy and concurrent chemoradiation. Eight of these 11 patients have since succumbed to progression of their disease. Of the p16-positive patients, 8/10 (80%) had a complete response. Of the p16-negative patients, 1/3 (33%) experienced a complete response.

### OS and PFS

**Figure 2:**
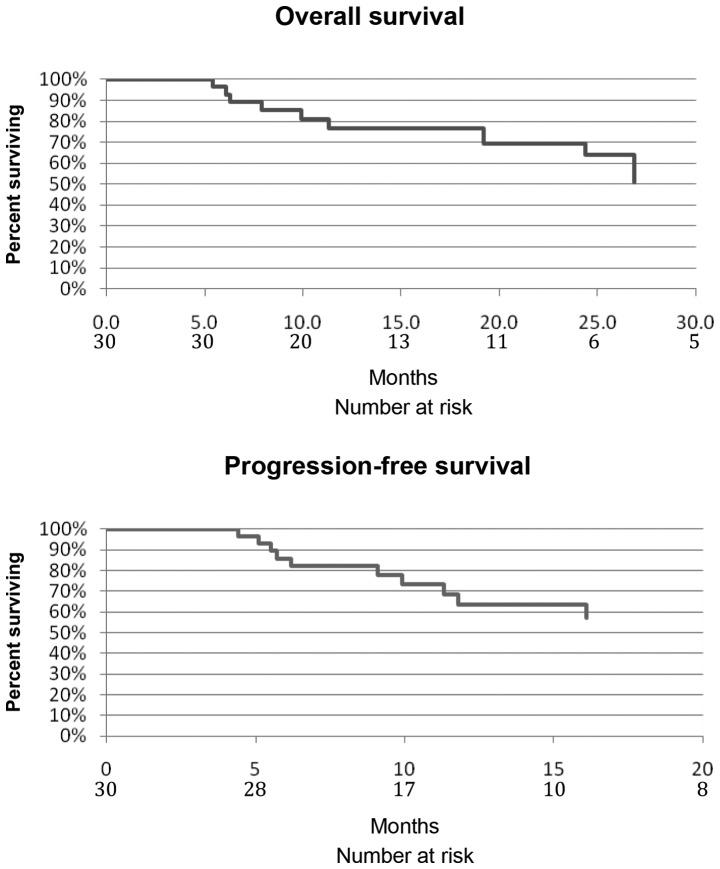
Overall and progression-free survival curves.

OS and PFS are still immature since median follow-up is only 13.7 months (range, 5.0–38.7 months). To date, 9 (30%) patients have experienced progressive disease and 9 (30%) patients have succumbed to disease. Of the 9 patients with progressive disease, 4 patients had a local recurrence (primary site; oral cavity, tonsil, supraglottic larynx, hypopharynx), 2 had metastatic recurrence (primary site; larynx, oral cavity) and 3 had local and metastatic recurrence (primary site; all three with tonsil). Of the 9 mortalities, 7 were attributed to disease progression. The eighth patient succumbed to unclear reasons after their last imaging examination demonstrated continued regression of disease. The ninth patient succumbed to injuries sustained in a motor vehicle accident while in a complete response (CR). Kaplan-Meier curves of PFS and OS are depicted in Fig. 2.

## Discussion

The TAX 323 and TAX 324 studies revealed the high toxicity and compromised feasibility of TPF-induction chemotherapy. While some patients may elect to accept short-term toxicities if they are associated with a higher rate of long-term cure, experience with this regimen has demonstrated that toxicity is capable of translating into long-term harm, with many patients either not able to proceed to definitive therapy or with severe compromises to planned chemoradiotherapy regimens. Furthermore, high toxicity of TPF has not translated into an increased response. As a result, the treating clinicians at the University of Pennsylvania switched from TPF to carboplatin/paclitaxel/cetuximab in May 2008, when induction chemotherapy was chosen.

The weekly regimen was well tolerated and the low rate of toxicity translated into excellent feasibility for the total treatment plan. The majority of patients received all intended cycles of induction chemotherapy, with high-dose intensity. The majority of patients’ body weights remained stable throughout induction, with 30% gaining in body weight. Grade 3 and 4 toxicity occurred in <7% of patients. Only a few hospitalizations occurred during induction and a high proportion of patients experienced relief of cancer pain and dysphagia. Additionally, all 30 patients were able to advance to definitive therapy with the majority able to receive the full, intended radiation dose on schedule and without treatment interruptions.

The excellent feasibility observed in our studies likely reflects the low toxicity of the induction regimen as well as the positive secondary effects from clinical responses. For patients restaged after induction chemotherapy, all but one patient had at least a partial response, with 30% obtaining complete response prior to initiation of chemoradiation. Relief of dysphagia and odynophagia likely resulted in the high rates of observed body weight stability, which in turn may have enabled our patients to more readily begin and complete definitive local-regional therapy. Definitive therapy in our cohort was fairly aggressive, as 74% of patients initiated definitive radiation with concurrent high-dose cisplatin. Of those patients, 70% completed their course with cisplatin (30% with other concurrent chemotherapy). As concurrent chemoradiation is the therapy best shown to increase cure rates compared with radiation alone (19), preservation of the intended treatment plan and the ability to receive the full dose of radiation likely contribute to overall outcomes.

Although still immature, our overall PFS and OS curves are inferior to those reported by Kies *et al* (16). This likely reflects the higher average stage of our patient population. In addition, to avoid bias, we included all patients receiving induction therapy, regardless of baseline PFS or other demographic variables. The higher average smoking history of our patient population may also contribute since tobacco history influences prognosis independent of HPV status (20).

Based on the extremely favorable outcomes reported by Kies *et al* for HPV+ patients, we chose to evaluate the treatment outcomes of our HPV^+^ patients. Although HPV data were only available for 13 of our patients (43%), the 80% complete RR observed in those with p16 positivity, in contrast to the 33% complete RR observed in those with p16 negativity, supports the findings of improved treatment response in the HPV-positive subset which has been previously reported (21–23).

In contrast to the high rates of treatment adherence reported in the present study, other previously published studies combining induction TPF with aggressive chemoradiation resulted in high toxicity and a considerably lower feasibility. For example, in the SWOG 0216 phase II study, cisplatin was used concurrently with definitive radiation following TPF induction (2). For the 74 patients in this trial, there was an 85% rate of grade 3–4 toxicity during induction chemotherapy as well as two mortalities during induction and a further two during subsequent chemoradiotherapy. Sixty-one patients (82.4%) completed induction chemotherapy and began concurrent chemoradiotherapy; 50 (68%) completed chemoradiotherapy. The design of the two major TPF trials conceded this limitation; in TAX 323 (6), no chemotherapy was added to definitive radiation and in TAX 324 (7), weekly, low-dose carboplatin (AUC 1.5) was administered. Even so, substantial toxicities and treatment delays during chemoradiation were observed in those receiving TPF induction.

The DeCIDE and PARADIGM trials sought to address whether the addition of induction chemotherapy to definitive chemoradiotherapy is able to improve survival. However, both trials were flawed at inception. In each trial, patients in the induction arms were treated with chemoradiotherapy regimens that are not generally considered as standard of care. Patients in DeCIDE received fluorouracil/hydroxyurea/docetaxel with split course radiotherapy and patients in PARADIGM received weekly carboplatin with standard XRT or weekly docetaxel with accelerated boost radiotherapy. No patient on the investigational arms received bolus cisplatin or cetuximab, the chemoradiotherapy regimens supported by phase III trial results and recommended by NCCN guidelines (11). Patients in the control arms also did not receive standard chemoradiotherapy. By contrast, in DeCIDE, they received the same regimen of split-course radiotherapy concurrent with fluorouracil/hydroxyurea/docetaxel used in the experimental arm and in PARADIGM, they received two (not the standard three) courses of cisplatin. Both studies terminated early due to poor accrual and also failed to meet their primary endpoints. In light of the negative results of the DeCIDE and PARADIGM trials, the feasibility and efficacy of the alternative weekly carboplatin/paclitaxel/cetuximab regimen outside of a clinical trial setting is of increased interest to the head and neck oncologist faced with clinical care decisions for the most locally advanced patients.

Despite the number of limitations associated with DeCIDE and PARADIGM, we nonetheless feel that the key problem was the choice of induction chemotherapy regimen. TPF is insufficiently active, too toxic and not feasible in combination with standard of care chemoradiotherapy. We believe that our off-protocol experience with carboplatin, paclitaxel and cetuximab lends support to the prospective experiences of Kies and Wanebo, that this regimen, outside of a formal clinical trial, is tolerable and very efficacious. As a result of this experience, we are prospectively studying induction chemotherapy regimens based on this platform. If our ongoing phase II study (24) is positive, we hope to evaluate induction chemotherapy in a phase III study in which both arms receive identical standard of care chemoradiotherapy.

## Figures and Tables

**Table I:** Patient and tumor characteristics (n=30).

Characteristic	Value
Age (years)	
Median	58.3
Range	37.4–83.6
Gender, n (%)	
Male	30 (100)
Female	0 (0)
Performance status, n (%)	
0	25 (83.3)
1	5 (16.7)
Charlson score, n (%)	
2	7 (23.3)
3	7 (23.3)
4	8 (26.7)
5	7 (23.3)
6	0 (0)
7	1 (3.3)
Smoking status, n (%)	
Never smokers	9 (30.0)
1–5 pk/year	8 (26.7)
6–20 pk/year	7 (23.3)
20–40 pk/year	3 (10)
>40 pk/year	3 (10)
Site of primary tumor, n (%)	
Base of tongue	15 (50)
Tonsil	8 (26.7)
Hypopharynx	1 (3.3)
Larynx	2 (6.7)
Oral cavity	4 (13.3)
HPV status, n (%)	
p16 status unknown	17 (56.7)
p16 positive	10 (33.3)
p16 negative	3 (10)

pk, pack; HPV, human papilloma virus.

**Table II:** Tumor (T) and nodal (N) distribution (n=30).

N	No. of patients by T classification					Total no. of patients
	Tx	T1	T2	T3	T4	
N0			1			1
N1						0
N2a			1		1	2
N2b	2		5	2	3	12
N2c			2	1	8	11
N3		1	1		2	4
Total	2	1	10	3	14	30

**Table III:** Induction chemotherapy.

Parameter	Carboplatin	Paclitaxel	Cetuximab
Number of doses administered			
Median	7	8	8
Range	2–8	2–8	0–8
Dose intensity, (%)			
Total doses expected	229	229	229
Total doses administered	211 (92.1)	212 (92.6)	195 (85.2)
Total doses held	18 (7.9)	17 (7.4)	34 (14.8)
Total doses reduced	0 (0)	21 (9.2)	2 (0.9)

Dose intensity represents the total number of doses for all patients delivered during all of induction.
